# Flexible Organic Thin Film Transistors Incorporating a Biodegradable CO_2_-Based Polymer as the Substrate and Dielectric Material

**DOI:** 10.1038/s41598-018-26585-0

**Published:** 2018-05-25

**Authors:** Cut Rullyani, Chao-Feng Sung, Hong-Cheu Lin, Chih-Wei Chu

**Affiliations:** 1 Department of Materials Science and Engineering, National Chiao Tung University, Hsinchu, 300 Taiwan (ROC); 2 Department of Photonics and Display Institute, National Chiao Tung University, Hsinchu, 300 Taiwan (ROC); 3 Research Center for Applied Science Academia Sinica, Taipei, 115 Taiwan (ROC); 4 College of Engineering, Chang Gung University, Taoyuan, 333 Taiwan (ROC)

## Abstract

Employing CO_2_-based polymer in electronic applications should boost the consumption of CO_2_ feedstocks and provide the potential for non-permanent CO_2_ storage. In this study, polypropylene carbonate (PPC) is utilized as a dielectric and substrate material for organic thin film transistors (OTFTs) and organic inverter. The PPC dielectric film exhibits a surface energy of 47 mN m^−1^, a dielectric constant of 3, a leakage current density of less than 10^−6^ A cm^−2^, and excellent compatibility with pentacene and PTCDI-C8 organic semiconductors. Bottom-gate top-contact OTFTs are fabricated using PPC as a dielectric; they exhibits good electrical performance at an operating voltage of 60 V, with electron and hole mobilities of 0.14 and 0.026 cm^2^ V^−1^ s^−1^, and on-to-off ratios of 10^5^ and 10^3^, respectively. The fabricated p- and n-type transistors were connected to form a complementary inverter that operated at supply voltages of 20 V with high and low noise margins of 85 and 69%, respectively. The suitability of PPC as a substrate is demonstrated through the preparation of PPC sheets by casting method. The fabricated PPC sheets has a transparency of 92% and acceptable mechanical properties, yet they biodegraded rapidly through enzymatic degradation when using the lipase from Rhizhopus oryzae.

## Introduction

Carbon dioxide (CO_2_), one of the most abundant substances on earth, is almost certainly responsible for the greenhouse effect that is increasing our atmosphere’s temperature^1^. Although CO_2_ is naturally present in the atmosphere as part of the earth’s carbon cycle, its release through human activity continues to rise and exceed the Nature’s ability to recycle it. Moreover, economic and industrial growth in developing countries is significantly increasing carbon emissions^2,3^. Indeed, 3.5 billion tons of CO_2_ is added annually, while the use of CO_2_ feedstocks is only approximately 3%^4^.

Interest in the use of CO_2_ as a raw material for chemical synthesis is growing; CO_2_ is abundant, inexpensive, non-flammable and renewable. In addition, preparing materials from CO_2_ not only decreases the atmospheric concentration of CO_2_ but also turns it into value-added products. One of the material products prepared from CO_2_ is polypropylene carbonate (PPC), a biodegradable aliphatic polyester synthesized from the copolymerization of CO_2_ and propylene oxide (PO) using a zinc complex as the catalyst^5,6^. This approach to producing a high yield of PPC is considered a green polymerization because no organic solvent is involved and no organic waste is produced^7^. PPC contains 44% CO_2_ by weight; it burns cleanly and gently in air without emitting harsh residues^8^. PPC can be tailored for applications with a broad range of material characteristics—from solid plastics to soft, flexible foams—depending on the length of the polymer chains. It can also be foamed and processed into thin films, and it readily mixes with other biopolymers^9,10^ or added organic/inorganic fillers^11,12^ to alter the thermal and mechanical properties.

In the past decade, flexible electronic and optoelectronic devices have piqued the interest of both industry and consumers because of their attractive properties, including light weight, bending ability, conformability, ruggedness, and rollability^13,14^. Flexible electronics that eschew rigid silicon-based semiconductors have been widely developed and used for many applications, including thin film transistors^15,16^, solar cells^17,18^, displays^19,20^, sensor arrays^21^, low-cost radio-frequency identification tags^22^, and logic circuits^23,24^. Nevertheless, with increasing growth of plastic electronics, the amount of solid plastic waste will probably increase dramatically, because the substrate that serves as the foundation for multiple functional layers occupies the largest part of such a device^25,26^. Biodegradable materials might be preferred alternatives that solve this problem. Various materials have been used as substrate and dielectric materials in the fabrication of green electronic devices, including synthetic polymers [e.g., polyvinyl alcohol^27^, poly(lactic acid)^28^, polycaprolactone^29^] and non-toxic and biodegradable materials obtained from Nature (e.g., paper^30^, chitin^31^, silk^32^, gelatin^33^, shellac^34^, collagen^35^, and cellulose-based polymers^36–39^).

In this study we explored the potential of PPC as a dielectric and substrate material for flexible electronics. We fabricated bottom-gate top-contact organic thin film transistors (OTFTs) and an organic inverter, each featuring PPC as the dielectric layer. We also prepared p- and n-type OTFTs on PPC substrate and evaluated their bending stability. Furthermore, we observed the rapid biodegradation of PPC substrates under enzymatic degradation using the lipase from *Rhizopus oryzae*. Employing CO_2_-based polymers for electronic applications should boost the use of CO_2_ feedstocks, lower the consumption of fossil fuel raw materials, and minimize electronic waste through biodegradability. Thus, our study has validated the reliability of PPC as a dielectric and substrate material for environmentally friendly thin film transistors.

## Results and Discussion

**Figure 1:**
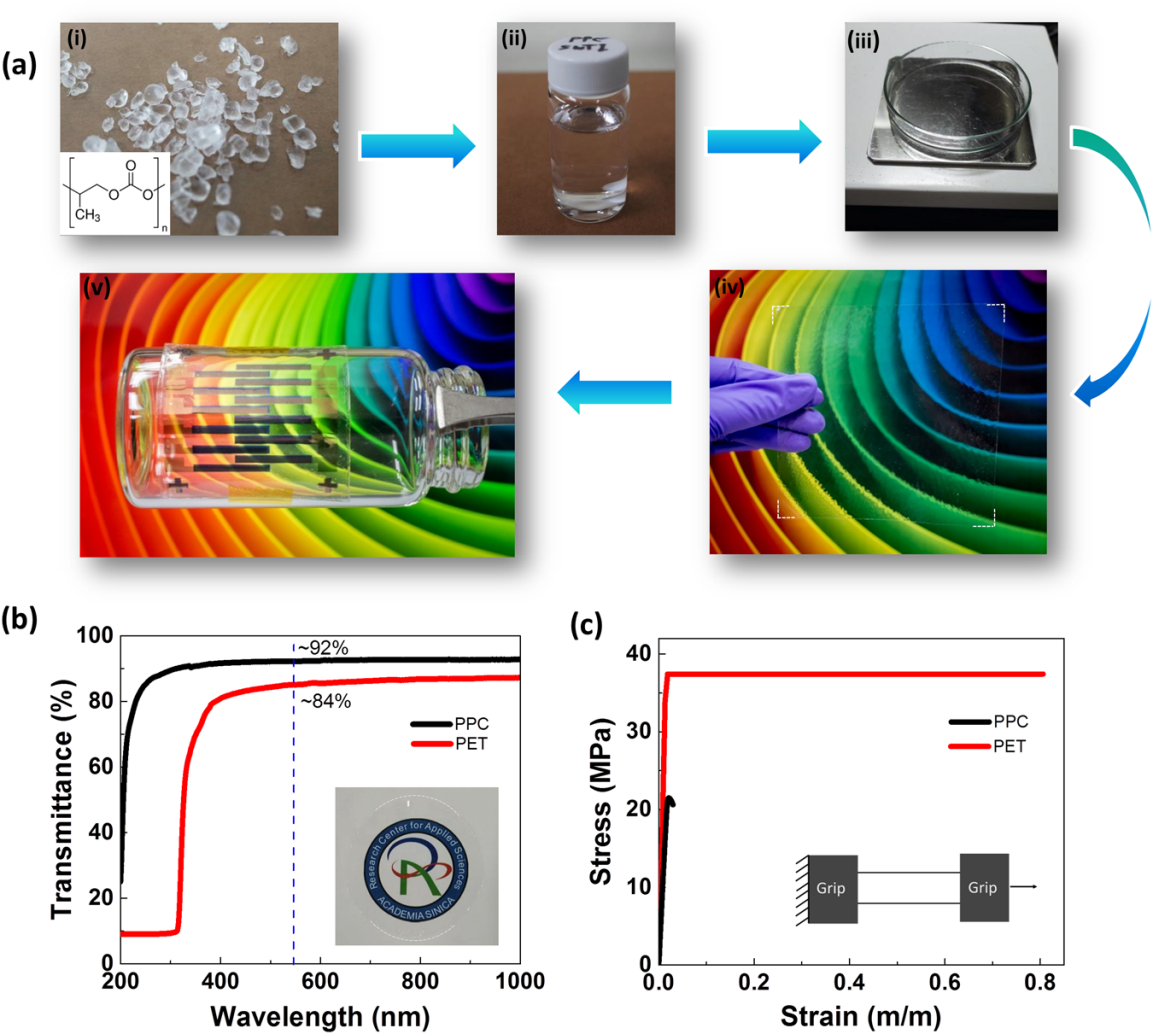
(**a**) General steps in the preparation of the PPC substrate through the casting method: (i) solid PPC pellets and its chemical structure; (ii) PPC dissolved in EtOAc; (iii) the solution is poured into a Petri dish and baked for 4 h; (iv) a 10 × 10 cm transparent PPC substrate; (v) OTFT device fabricated on the PPC substrate. (**b**) Transmittance spectrum of a 90-μm-thick PPC substrate. (**c**) Stress–strain curve obtained from tensile tests of PPC strips.

Figure 1a presents the general steps used to prepare the PPC substrates. The solid PPC pellets were dissolved in ethyl acetate (EtOAc); the solution was cast into a 4-inch Petri dish and baked to evaporate the solvent. The PPC sheet was readily detached from the Petri dish; after drying it was ready for use in device fabrication. The fabricated PPC substrates had excellent transparency in the visible (Fig. 1b); for example, the transmittance was approximately 92% at 550 nm for a 90-µm-thick PPC film. Mechanical stability of a substrate is an essential factor for functional and reliable electronics. From a structural point of view, PPC can be regarded as a flexible ductile polymer, due to the flexible carbonate linkages in the repeating unit^40^. Our PPC possessed an elastic modulus of 1.09 ± 0.04 GPa and an ultimate tensile stress of 19 ± 2.0 MPa, as extracted from the stress–strain data in Fig. 1c. Although these values are lower than those of commercial polyethylene terephthalate (PET) substrates or previously reported cellulose-based substrates, they are sufficient for the fabrication of flexible OTFT devices displaying good electrical performance. As previously reported, thin substrate with low modulus exhibits better flexibility and reduces the strain level in the device layer^41,42^.

**Figure 2:**
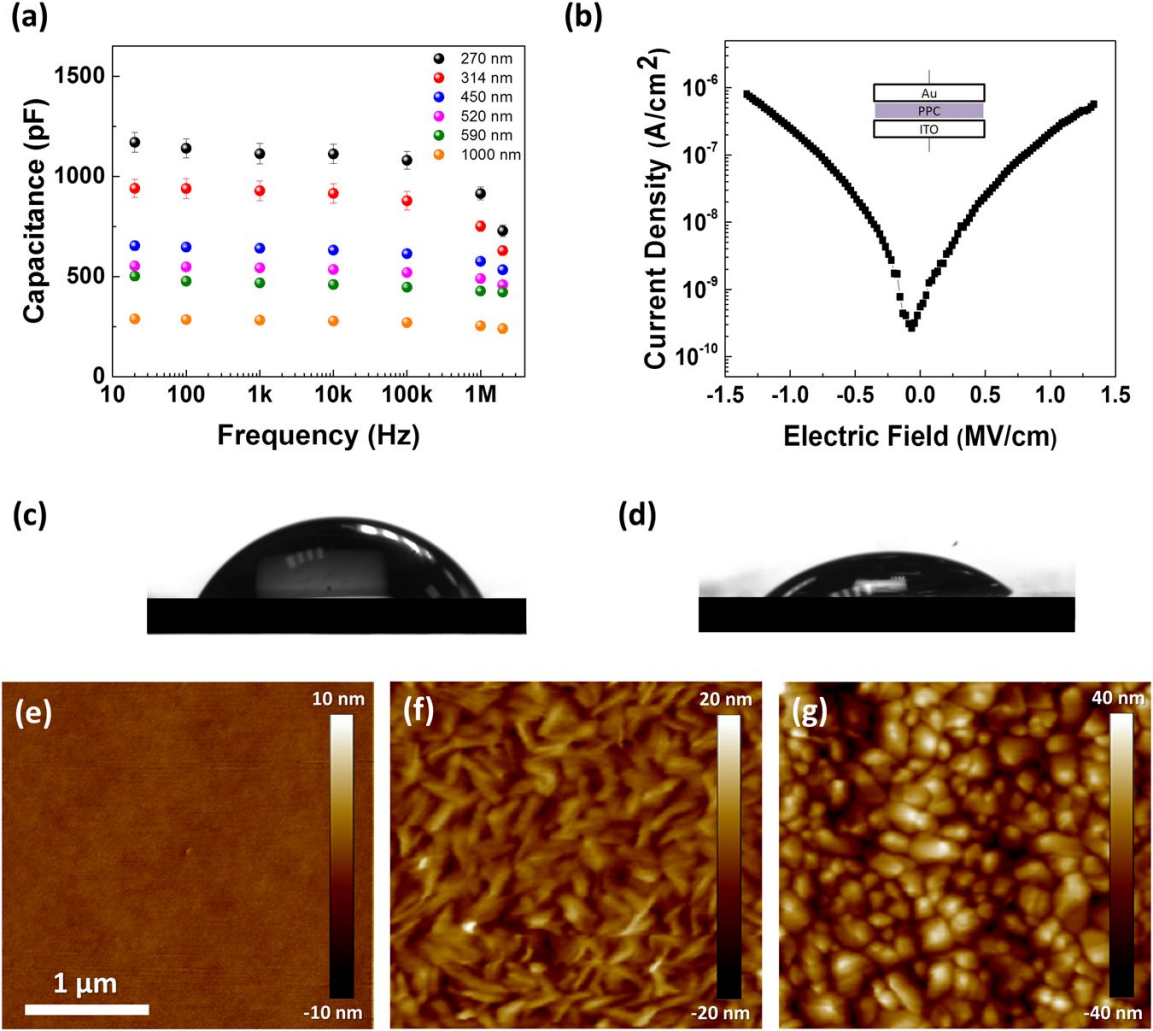
(**a**) Capacitance of the PPC at various film thicknesses, measured at frequencies from 20 Hz to 2 MHz. (**b**) Leakage current density of a 450-nm-thick PPC dielectric, measured under electric field strengths from −1.5 to 1.5 MV cm^−1^. (**c**) Water and (**d**) diiodomethane contact angles of the PPC film. (**e**–**g**) AFM images (3 µm × 3 µm) of (**e**) the PPC film on ITO/glass and (**f**) PTCDI-C8 and (**g**) pentacene films grown on the PPC dielectric.

Here, we explored PPC not only as a substrate but also as a dielectric. To evaluate its performance as a gate dielectric material, we fabricated indium tin oxide (ITO)/PPC/Au sandwich structure capacitors and determined their capacitance, dielectric constant, and leakage current density. Figure 2a presents the capacitance of PPC films of various thicknesses, measured in the frequency range from 20 Hz to 1 MHz. The dielectric constant, derived from the capacitance data, was 3, consistent with previously reported values^43^. We evaluated the leakage current of a 450-nm-thick PPC film using the same metal-insulator-metal capacitor structure. Figure 2b reveals that the leakage current density increased upon increasing the electric field. The PPC film yielded a leakage current density of less than 1.0 × 10^−6^ A cm^−2^ at electric field strengths from −1.5 to 1.5 MV cm^−1^.

The surface properties of dielectric films will impact the growth of small-molecule organic semiconductors because they affect the overall device performance. Dielectric roughness reduces the charge transport mobilities in organic semiconductors; The surface energy impacts the performances of OTFTs by influencing the orientation of semiconductor molecules and the morphology of semiconductor layers^44^. Therefore, we measured contact angles and conducted AFM experiments to study the surface energy and morphology of PPC as well as those of layered organic semiconductors grown on PPC dielectrics. For a spin-coated PPC film, contact angle measurements using DI water gave a value of surface energy (γ) of 72.8 mN m^−1^ [separable into a polar component (γ_p_) of 51.8 mN m^−1^ and a dispersive component (γ_d_) of 21.8 mN m^−1^)]; for diiodomethane, this value was 50.8 mN m^−1^ [separable into a polar component (γ_p_) of 0 mN m^−1^ and a dispersive component (γ_d_) of 50.8 mN m^−1^]. The surface of PPC was hydrophilic, with contact angles of DI water and diiodomethane of 69 and 42°, respectively (Fig. 2c and d). The total surface energy, calculated using the geometric mean method, was 47 mN m^−1^, slightly lower than the lowest surface energy of pentacene crystal planes (48 mN m^−1^)^45^. The AFM image in Fig. 2e reveals a uniform and smooth surface morphology for the PPC film. The measured root-mean-square (RMS) surface roughness of the PPC film was 0.29 nm. Figure 2f and g present AFM images of N,N′-Dioctyl-3,4,9,10 perylenedicarboximide (PTCDI-C8) and pentacene films deposited on the PPC. The PTDCI-C8 film featured typical rod-like grains, similar to those grown on other polymer dielectrics^46^; its surface roughness was 6.31 nm. In contrast, the pentacene film on the PPC featured small grains, instead of its typical dendritic grains; the surface roughness was 6.73 nm. The small pentacene grains were formed through a Volmer–Weber growth mode that led to the formation of three-dimensional islands; this phenomenon occurs when the deposited molecules are more strongly bound to each other than to the underlying film, because the surface energy of the dielectric film is lower than that of pentacene^47^.

**Figure 3:**
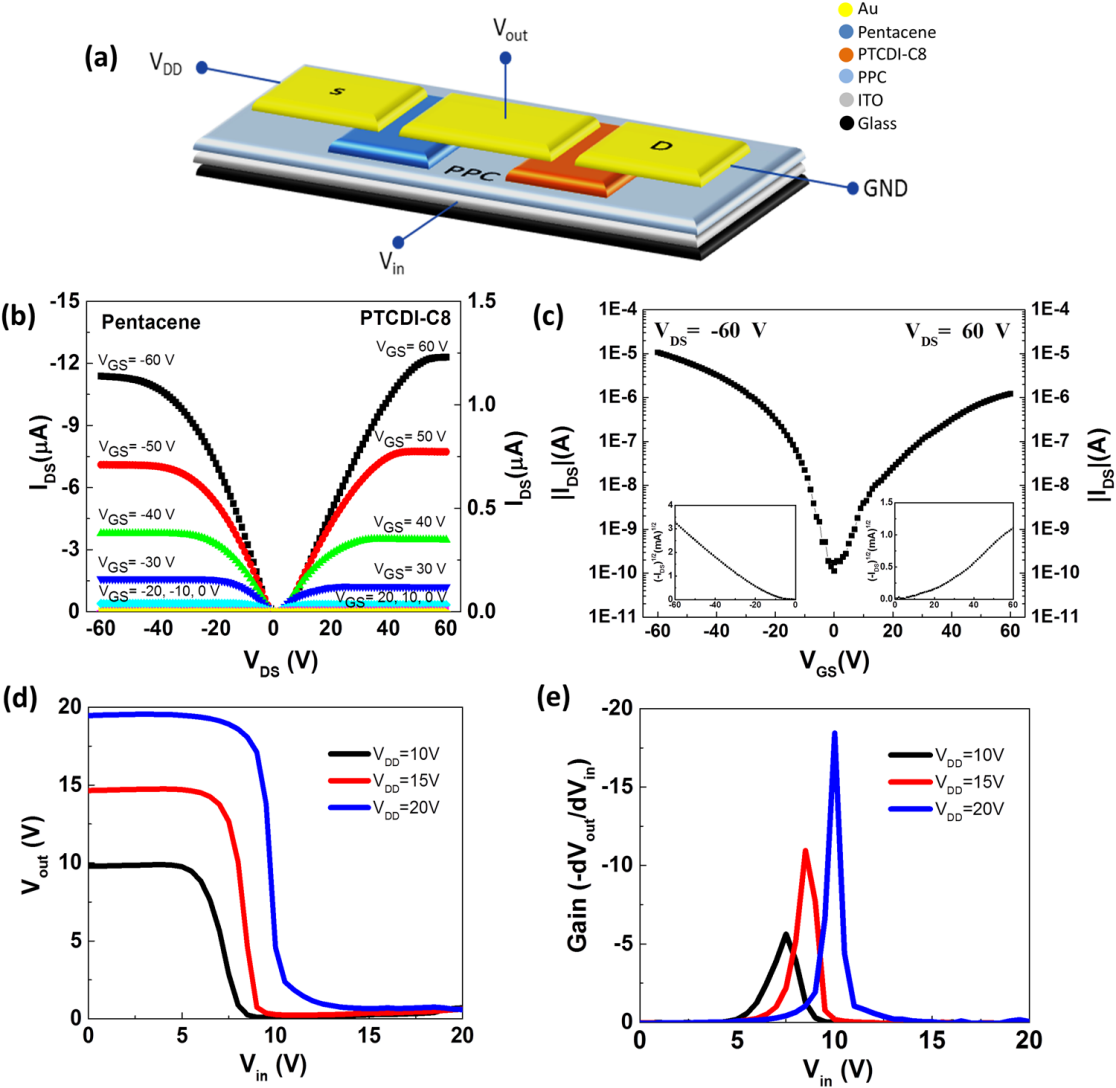
(**a**) Schematic representation of pentacene and PTCDI-C8 OTFTs coupled to form a complementary organic inverter. (**b**) Output and (**c**) transfer characteristics of pentacene and PTCDI-C8 OTFTs featuring 450-nm-thick PPC as the dielectric. (**d**) Voltage transfer characteristics and (**e**) gain of the complementary inverter plotted with respect to the input voltage (*V*_in_), when operated at values of *V*_DD_ of 10, 15, and 20 V.

Figure 3a provides a schematic representation of a p- and n-type transistor coupled to form an inverter. The devices featured an ITO gate, a PPC dielectric, a pentacene or PTCDI-C8 channel, and a MoO_3_/Al (for the pentacene OTFT) or Au (for the PTCDI-C8 OTFT) electrode. Figure 3b and c display the output and transfer characteristics of the fabricated pentacene and PTCDI-C8 OTFTs. The device performances are summarized in Table 1. The pentacene OTFT using Au as the source drain exhibited a charge carrier mobility of 0.138 ± 0.007 cm^2^ V^−1^ s^−1^, a threshold voltage (*V*_th_) of −19.17 ± 0.85 V, and an on-to-off ratio of 10^4^ (Supplementary Fig. S1). The performance of the pentacene OTFT improved after replacing the Au electrode with the MoO_3_/Al bilayer electrode. Although aluminum is a well-established, inexpensive electrode material displaying good corrosion resistance, its low work function limits its applicability as an electrode material for p-type OTFTs. The performance of OTFTs can be enhanced by inserting a MoO_3_ layer between the Al electrode and the organic semiconductor^48–50^. MoO_3_ is a wide-gap semiconductor having a band gap of 3–3.1 eV and an electron affinity of approximately 2.2 eV, implying a valance band position near 5.3 eV. The highest occupied molecular orbital (HOMO) of pentacene lies at 5.0 eV and is aligned with the valence band of MoO_3_, resulting in no barrier for injection of holes into the pentacene layer^50^. The pentacene OTFT featuring the MoO_3_/Al electrode exhibited a charge carrier mobility of 0.142 ± 0.004 cm^2^ V^−1^ s^−1^, a value of *V*_th_ of −15.63 ± 2.24 V, and an on/off ratio of 10^5^. The PTDCI-C8 OTFT featuring the Au electrode displayed a charge mobility of 0.026 ± 0.002 cm^2^ V^−1^ s^−1^, a value of *V*_th_ of 19.32 ± 2.57 V, and an on-to-off ratio of 10^3^. To form a symmetrical device structure, we attempted to use a MoO_3_/Al electrode for the PTCDI-C8 device, but the charge mobility dropped as a result of a mismatch in the energy levels of the HOMO and lowest unoccupied molecular orbital (LUMO), and significant shift in threshold voltage occurred to the positive side; as a result, the inverter performance was unbalanced. We fabricated a complementary inverter by connecting the pentacene device (with MoO_3_/Al electrode) and the PTCDI-C8 device (with Au electrode). The complementary inverter exhibited good switching behavior and voltage amplification at supply voltages (*V*_DD_) of 10–20 V, as revealed in voltage-transfer characteristics (VTC) of the organic inverter in Fig. 3(d). We calculated the noise margins at high (NMH, corresponding to logic 1) and low (NML, corresponding to logic 0) levels using the equations

1 $${N}_{MH}={V}_{outH}-{V}_{inH}$$

2 $${N}_{ML}={V}_{inL}-{V}_{outL}$$

**Table 1:** Summary of OTFT performance with different substrate and dielectric layers. All values are based on performance of 6 different samples.

Substrate	Dielectric	Semiconductor	Source Drain	µ [cm^2^ V^−1^ s^−1^]	Vth [V]	On-Off Ratio
ITO coated glass	PPC	Pentacene	MoO3/Al	0.142 ± 0.004	−15.63 ± 2.24	10^5^
ITO coated glass	PPC	PTCDI	Au	0.026 ± 0.002	19.32 ± 2.57	10^3^
PPC	PVPy	Pentacene	MoO3/Al	0.27 ± 0.02	−15.72 ± 0.36	10^4^
PPC	PVPy	PTCDI	Au	0.28 ± 0.02	12.44 ± 0.65	10^4^

The NMH and NML were 85 and 69% of the maximum value of 1/2 *V*_DD_, suggesting good noise immunity for the fabricated inverter device. The signal inverter gain, defined as the maximum value of the slope of the transfer curve, increased upon increasing the value of *V*_DD_, as revealed in Fig. 3(e). A gain of 18 was achieved when the organic complementary inverters were operated at a value of *V*_DD_ of 20 V.

**Figure 4:**
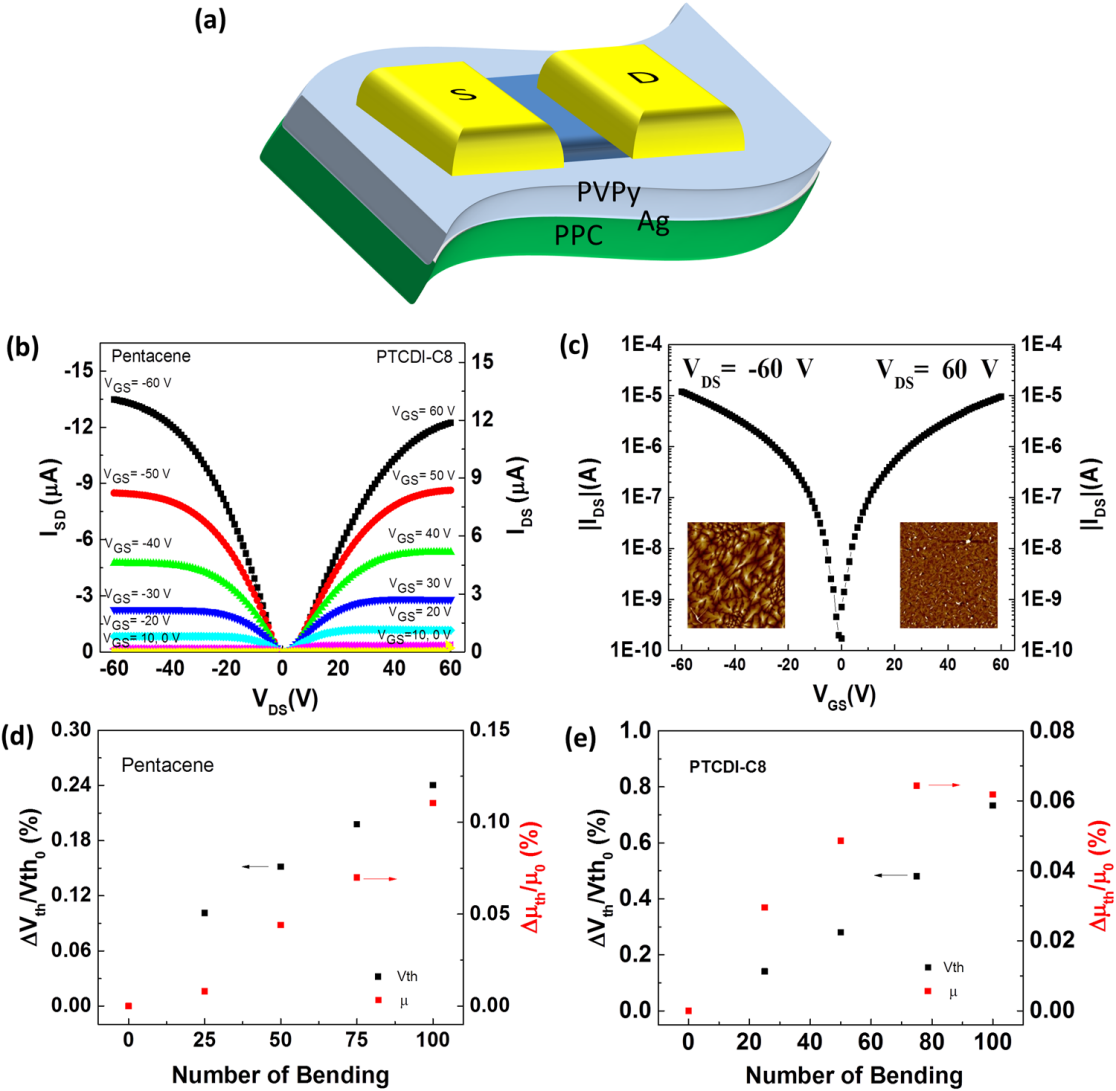
(**a**) Schematic representation of a bottom-gate top-contact OTFT on the PPC substrate. (**b**) Output and (**c**) transfer characteristics of pentacene and PTCDI-C8 OTFTs fabricated on the flexible PPC substrate. Inset: Corresponding AFM images of pentacene and PTCDI-C8 semiconductors grown on a PVPy dielectric. (**d**,**e**) Corresponding *V*_th_ shifts and mobility changes of (**d**) pentacene and (**e**) PTCDI-C8 OTFTs after 100 cycles of bending at a radius of 1.5 cm.

To demonstrate the suitability of using PPC also as a substrate, we fabricated pentacene and PTCDI-C8 OTFTs on PPC substrates and evaluated their bending stability (Fig. 4a). A silver film of 25 nm was deposited on the PPC surface to form a metal gate prior to fabrication of these OTFT devices. PPC has a low glass transition temperature (*T*_g_ = ca. 40 °C) and limited solvent-resistance, limiting the choice of dielectric materials available for device fabrication. We found, however, that PPC had good resistance to water and alcohol-based solvent (e.g., isopropyl alcohol (IPA), ethanol (EtOH), methanol (MeOH)), but was damaged when exposed to chloroform, propylene glycol methyl ether acetate (PGMEA), acetone, and toluene. We chose polyvinylpyrrolidone (PVPy) for the dielectric layer for the following reasons: (i) PVPy is a biocompatible polymer with low environmental toxicity; (ii) PVPy is soluble in MeOH, and the PPC substrate had good resistance to MeOH; and (iii) MeOH has a low boiling point, so the solvent could be evaporated without heating the substrate at too high a temperature. A 970-nm-thick layer of PVPy was spin-coated as the dielectric; it also functioned as a planarization layer for improved organic semiconductor growth. Figure 4b and c present the output and transfer characteristics of pentacene and PTCDI-C8 OTFTs fabricated on the PPC substrate. The pentacene OTFT featuring a MoO_3_/Al electrode exhibited a charge carrier mobility of 0.27 ± 0.02 cm^2^ V^−1^ s^−1^, a value of *V*_th_ of −15.72 ± 0.36 V, and an on/off ratio of 10^4^ at gate voltage of 60 V; for the PTCDI-C8 OTFT featuring a Au electrode, these values were 0.28 ± 0.02 cm^2^ V^−1^ s^−1^, 12.44 ± 0.65 V, and 10^4^, respectively. The mobilities of pentacene and PTCDI-C8 OTFTs fabricated on PPC substrate are higher in contrast to OTFTs with similar structure and operating voltage reported previously, which are in the range of 0.05–0.15 cm^2^ V^−1^ s^−1^ for pentacene^51,52^ and 0.02–0.015 cm^2^ V^−1^ s^−1^ for PTCDI-C8^46^. The inset to Fig. 4c displays AFM images of pentacene and PTCDI-C8 semiconductors grown on the PVPy dielectric. We evaluated the flexibility of the devices fabricated on the PPC substrate through cyclic bending at a radius of 1.5 cm. Figure 4d and e plot the values of *V*_th_ and the mobility changes of pentacene and PTCDI-C8 OTFTs with respect to the number of bending cycles. The devices exhibited no significant shifts in their transfer curves, as indicated by the increasing values of *V*_th_ of 0.24% for the pentacene OTFT and 0.73% for the PTCDI-C8 OTFT after 100 bending cycles. In addition, we observed 0.11 and 0.06% increases in mobility for the pentacene and PTCDI-C8 OTFTs, respectively.

**Figure 5:**
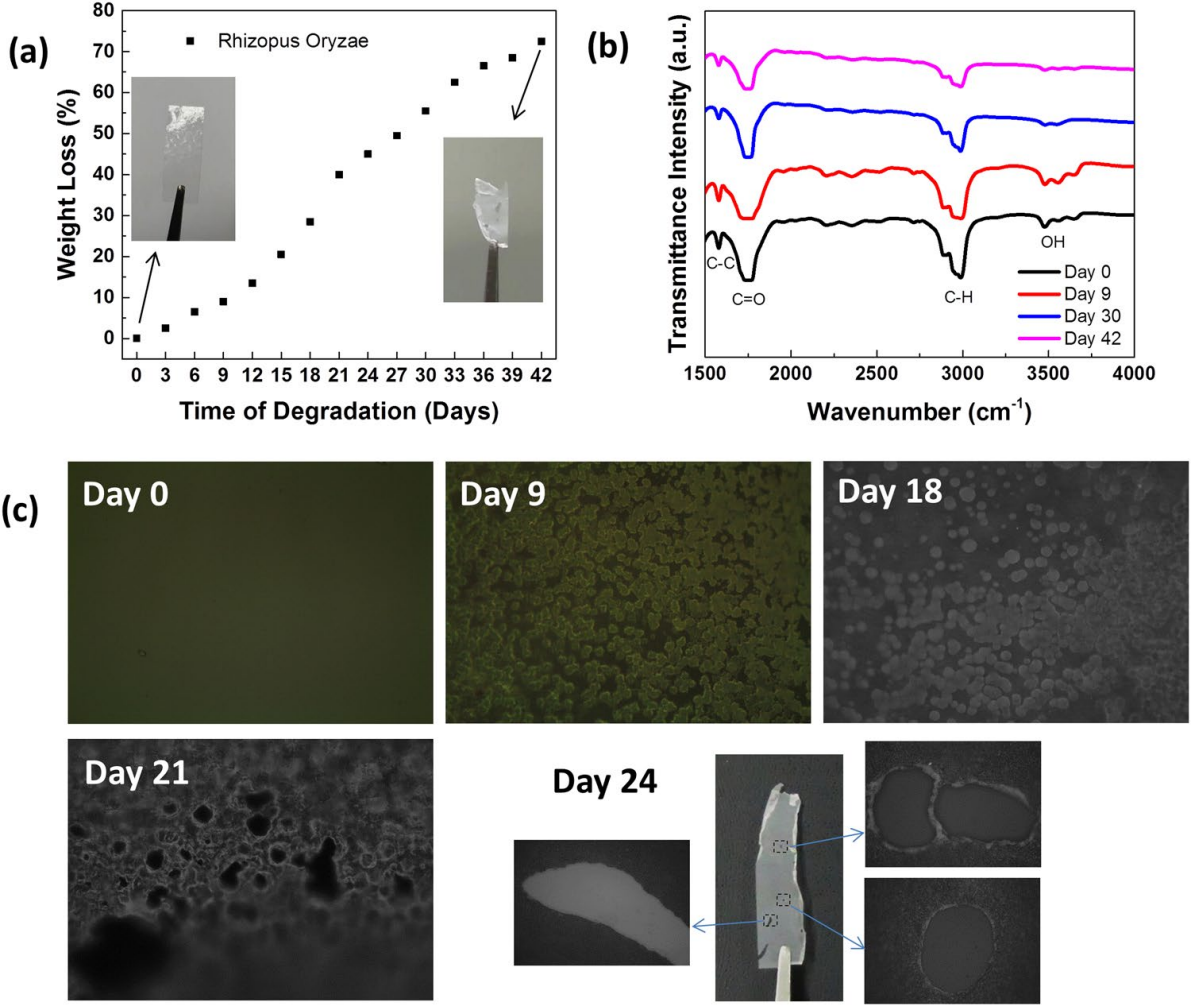
(**a**) Degradation of the PPC film mediated by the lipase from *Rhizhopus oryzae* in a buffer solution (pH 7.0) at 37 °C for 42 days. (**b**) FTIR spectra and (**c**) optical images of the PPC film before and after immersion in the solution of the lipase from *Rhizhopus oryzae* for 9, 18, 21, and 24 days.

Previous studies of the biodegradation of PPC in air, soil, and water and its hydrolysis and behavior in a weathering chamber have suggest that decomposition can be reached, but only slightly and dependent on the test conditions^43,53^. In this study, we examined the degradation of PPC through enzymatic degradation using a lipase, which has been reported to degrade polymers such as poly(4-hydroxyalkanoate)^54^ and polycaprolactone^55^. We determined the extent of PPC degradation by lipase by measuring its weight loss after intervals of 3 days. The weight of each test specimen was measured before and after composting; the weight-loss degradability of the film was calculated from the change in weight of each test piece during composting. The weight losses after the first 3 days of immersion in a solution of the lipase from *Rhizopus oryzae*, the lipase from porcine pancreas, and the lipase from *Candida rugosa* were 2.50, 1.50, and 1.19%, respectively. In a blank experiment using only buffer solution (i.e., no enzymes), we observed no degradation of the PPC; the sample weight and color did not change after immersion in the buffer solution, even after 12 days. The degradability of PPC using the lipase from *Rhizopus oryzae* was the highest among the enzymes tested. Supplementary Fig. S2 compares the weight losses induced by these three enzymes after 12 days of treatment. The weight loss of the PPC in the *Rhizopus oryzae* solution increased upon increasing the immersion time: 13.5% weight loss after 12 days and 72.5% after 42 days (Fig. 5a).

We recorded Fourier transform infrared (FTIR) spectra of the PPC before and after immersion in the *Rhizopus oryzae* solution, to evaluate the structural changes that occurred during the enzymatic degradation. The FTIR spectrum of the pristine PPC featured several characteristic peaks: a small peak at 1580 cm^−1^, presumably corresponding to carboxylate groups present at the ends of the polymer chains; a strong peak at 1740 cm^−1^, attributed to the stretching of C=O bonds; a broad peak at 3000 cm^−1^, representing C–H bond stretching in the propylene units; and a small peak at 3500 cm^−1^, presumably for the terminal OH groups^56–58^. The FTIR spectra of the PPC that had been treated for 9, 30, and 42 days revealed almost no peak shifts, but they all featured lower peak intensities, compared with the spectrum of the pristine PPC. Decreasing intensities of characteristic peaks is an indication of the degradation of polymer films^59–63^. In this case, the decreases in intensity of the C = O, C–H, and O–H bands are consistent with lipase attack on the ester bonds of the PPC. The peak intensities decreased continuously upon increasing of treatment time (Fig. 5b).

Optical microscopy revealed the changes on the surface of the degraded PPC. Figure 5c presents optical images of the PPC samples before and after treatment with the *Rhizopus oryzae* solution. The untreated PPC had a very smooth surface without any significant defects. During the early days of treatment, no substantial changes occurred other that the sample color slightly turning whitish with decreased transparency. After 9 days of treatment, morphological changes were evident. The sample color became whitish and the film became more fragile and thinner. The surface of the PPC became rough and non-uniform, an indication of surface erosion. Similar phenomena and a larger coverage area of erosion occurred until day 18 of treatment. On day 21, pits and cavities appeared on the PPC surface; bigger holes with diameters of a few hundred micrometers were observed on day 24.

The biodegradation mechanism was presumably similar to that generally reported for the biodegradation of plastic^64^. The initial attack begins with the adherence of enzymes onto the PPC surface; in this case, quite favorably, because the PPC surface is hydrophilic. The next step is the breaking of ester bonds mediated by the lipase from *Rhizopus oryzae*, followed by erosion of the PPC surface. The process continues through formation of cavities and pits on the PPC surface, due to continued enzymatic digestion. Moreover, the incubation occurred at 37 °C, close to the value of *T*_g_ of the PPC (i.e., the temperature at which the amorphous parts of the polymer become flexible and more accessible to enzymatic attack)^65^. Biodegradation by enzyme is a promising method to be used in the decomposition of PPC at a large scale, but some better preservation ways of enzymes are necessary to keep their effective activities for a longer time.

## Conclusions

We have demonstrated the suitability of using PPC as a dielectric material and a flexible substrate for electronic applications. A 450-nm-thick PPC film exhibited a dielectric constant of 3 and a leakage current density of less than 10^−6^ A cm^−2^. Pentacene and PTCDI-C8 OTFTs employing PPC as the dielectric displayed charge carrier mobilities of 0.14 and 0.026 cm^2^ V^−1^ s^−1^, respectively, with on/off ratios of 10^5^ and 10^4^, respectively. A corresponding organic inverter exhibited an NMH of 85% of 1/2 *V*_DD_, an NML of 69% of 1/2 *V*_DD_, and a maximum gain of 18 at a value of *V*_DD_ of 20 V. The PPC substrate prepared by casting of a PPC solution exhibited very high transparency, an elastic modulus of 1.09 GPa, and an ultimate tensile stress of 19 MPa. Pentacene and PTCDI-C8 OTFTs fabricated on the PPC substrate displayed remarkable electrical performance, with carrier mobilities of 0.27 and 0.28 cm^2^ V^−1^ s^−1^ and each with an on/off ratio of 10^4^. Degradation of the PPC substrate mediated by the lipase from *Rhizopus oryzae* occurred with a weight loss of 72.5% after 42 days of treatment. Accordingly, PPC appears to be very suited for use in the preparation of inexpensive, environmentally friendly OTFTs. Nevertheless, further study will be needed to improve the mechanical strength and low thermal stability of PPC for broader application.

## Methods

### Preparation and characterization of PPC substrate

5 wt% of PPC (*M*_n_ = ca. 50,000; Sigma–Aldrich) was dissolved in EtOAc and stirred overnight. The solution was poured into a 4-inch Petri dish and baked at 110 °C for 4 h to evaporate the solvent. Transmittance spectra of the formed PPC sheet were measured in the wavelength range 200–1000 nm using a Halo RB-10 Dynamica Spectrophotometer. Stress-strain curve was obtained by horizontally stretching a PPC strip (dimensions: 1 × 5 cm) using an MTS Tytron 250 pull tester and obeying ASTM D882. Young’s modulus was determined from the slope of linear region of stress strain curve.

### Fabrication and characterization of PPC dielectric

ITO/PPC/Au capacitors were fabricated featuring a PPC layer sandwiched between ITO bottom and Au top electrodes. The PPC solution was spin-coated at various spin speed to vary the film thickness. To prepare thicker films, 8 wt% PPC solution was spin coated at 1000 and 3000 rpm to obtain 1000 and 590 nm, respectively. As for thinner films, 5 wt% PPC solution was spin coated at 800, 1000, 2000 and 3000 rpm to acquire 520, 450, 314, and 270 nm PPC film, respectively. PPC film thicknesses were measured using a VEECO Detak surface profilometer. The capacitance value was measured in parallel circuit mode (Cp-D) using an Agilent E4980A Precision LCR meter at frequencies in the range from 20 Hz to 2 MHz. The active area of the capacitor for capacitance and leakage current measurement was 0.1 cm^2^. The dielectric constant was calculated using the equation

3 $$k=\frac{Cd}{{{\rm{\varepsilon }}}_{0}{\rm{A}}}$$

where *C* is the capacitance, *ε*_0_ is the permittivity of a vacuum, *d* is the thickness of the film, and *A* is the active area of the capacitor. Contact angles of water and diiodomethane on the PPC surfaces were measured using a Magicdroplet model 100 goniometer. Surface energy was calculate from contact angle data using geometric mean method

4 $$\sqrt{{\gamma }_{s}^{d}{\gamma }_{l}^{d}}+\sqrt{{\gamma }_{s}^{p}{\gamma }_{l\,}^{p}}={\gamma }_{l}(cos\theta +1)$$

where γ_sl_ is the coresponding surface energy of the solid-liquid interface, γ_s_ is surface energy of solid, γ_l_ is surface energy of measuring liquid, and ${\gamma }_{s}^{d},{\gamma }_{l}^{d},$ ${\gamma }_{s}^{p},{\gamma }_{l}^{p}\,$are the dispersion and polar components. Surface energy of water was 72.8 mN m^−1^, separable into a polar component of 51.8 mN m^−1^ and a dispersive component of 21.8 mN m;^−1^ for diiodomethane, this value was 50.8 mN m^−1^, separable into a polar component of 0 mN m^−1^ and a dispersive component of 50.8 mN m^−1^.

Film morphologies were obtained using a Bruker dimension icon atomic force microscope operated in tapping mode.

### Fabrication and characterization of OTFTs and organic inverter

Pentacene and PTCDI-C8 OTFTs were fabricated in a bottom-gate top-contact configuration on ITO coated glass substrates. First, 5 wt% PPC in EtOAc was spin-coated (1000 rpm) on the ITO coated glass substrate and baked at 90 °C for 20 min to achieve a thickness of 450 nm. After spin-coating of the PPC dielectric layer, 70-nm pentacene and 60-nm PTCDI-C8 organic layers were thermally evaporated under vacuum at a deposition rate of 0.4 A s^−1^, followed by deposition of a 10-nm MoO_3_/40-nm Al (for the pentacene device) or 40-nm Au (for the PTCDI-C8 device) source-drain electrode through a shadow mask (channel length: 200 μm; channel width: 2000 μm). The fabricated pentacene and PTCDI-C8 OTFTs were coupled to form an organic inverter. For fabrication of flexible OTFTs on PPC substrates, the PPC sheet was cut into pieces (dimensions: 2 × 2 cm) and attached to a glass support to simplify the fabrication process. A 25-nm-thick Ag metal gate was deposited on the PPC substrate, followed by spin-coating (4000 rpm) of 7 wt% of PVPy in MeOH, then baking at 35 °C for 1 h. The film thickness of spin-coated PVPy was 970 nm. Next, 70-nm pentacene and 60-nm PTCDI-C8 organic semiconductors were thermally evaporated, followed by deposition of a 10-nm MoO_3_/40 nm Al or 40-nm Au source-drain electrode. MoO_3_, Al, and Au gates were thermally evaporated under vacuum at pressure of 5.0 × 10^−6^. All current–voltage measurements were performed using a Keithley 4200-SCS parameter analyzer in a N_2_ glove box.

The threshold voltage was extracted from the square root of drain current and the field-effect mobility of OTFT was calculated using the equation

5 $${{\rm{I}}}_{D}=(\frac{W}{2L}){C}_{i}\mu {({V}_{G}-{V}_{T})}^{2}\,({\rm{for}}\,{\rm{the}}\,{\rm{saturated}}\,{\rm{regime}})$$

where *I*_D_ is the drain current in the saturated regime; *V*_G_ and *V*_T_ are the gate and threshold voltages, respectively; *W* and *L* are the channel width and length, respectively; *C*_i_ is the capacitance per unit area of the gate dielectric layer; and *µ* is the field-effect mobility.

### Degradation study

Lipase from *Rhizopus oryzae*, lipase from porcine pancreas, and lipase from *Candida rugosa* were purchased from Sigma–Aldrich. Enzymatic degradation was performed by placing each lipase (4 mg) in 0.02 M phosphate buffer solution (pH 7.0) at 37 °C. The PPC sheet with thickness of 50 µm was cut into rectangular shapes (dimensions: 1 × 5 cm), weighed, and then immersed in the enzyme solution. The specimens were removed every 3 days, rinsed with distilled water, dried with tissue paper, dried under vacuum for 1 h, and then weighed. The enzyme solutions were replaced every 3 days to preserve the enzyme activity (i.e., the enzymes had limited lifetime). Optical images were recorded using an Olympus BX51 microscope equipped with a camera. The weight losses were determined using the formula

6 $$Weigth\,loss=\frac{Initial\,weight-Final\,weight}{Initial\,weight}\times 100 \% $$

FITR spectra were recorded using a Bruker Vertex 70 V spectrometer operated in absorbance mode.

## Electronic supplementary material

Supplementary information
