# Supplementary material for: Flexible Organic Thin Film Transistors Incorporating a Biodegradable CO2-Based Polymer as the Substrate and Dielectric Material
Source: Sci Rep. 2018 May 25;8:8146. doi: 10.1038/s41598-018-26585-0 (PMC5970150; doi:10.1038/s41598-018-26585-0)
Supplement: Supplementary file 1 — Supplementary information [file 41598_2018_26585_MOESM1_ESM.docx]

Supporting information

**Flexible Organic Thin Film Transistors Incorporating a Biodegradable CO_2_-Based Polymer as the Substrate and Dielectric Material**

Cut Rullyani^a^, Chao-Feng Sung^b^, Hong-Cheu Lin^a^* and Chih-Wei Chu^c,d^*

^a^ Department of Materials Science and Engineering, National Chiao Tung University, Hsinchu, 300, Taiwan (ROC); E-mail: [linhc@mail.nctu.edu.tw](mailto:linhc@mail.nctu.edu.tw)

^b^ Department of Photonics and Display Institute, National Chiao Tung University, Hsinchu 300, Taiwan (ROC)

^c^ Research Center for Applied Science Academia Sinica, Taipei, 115, Taiwan (ROC); E-mail: [gchu@gate.sinica.edu.tw](mailto:gchu@gate.sinica.edu.tw)

^d^ College of Engineering, Chang Gung University, Tao-Yuan 333, Taiwan (ROC)


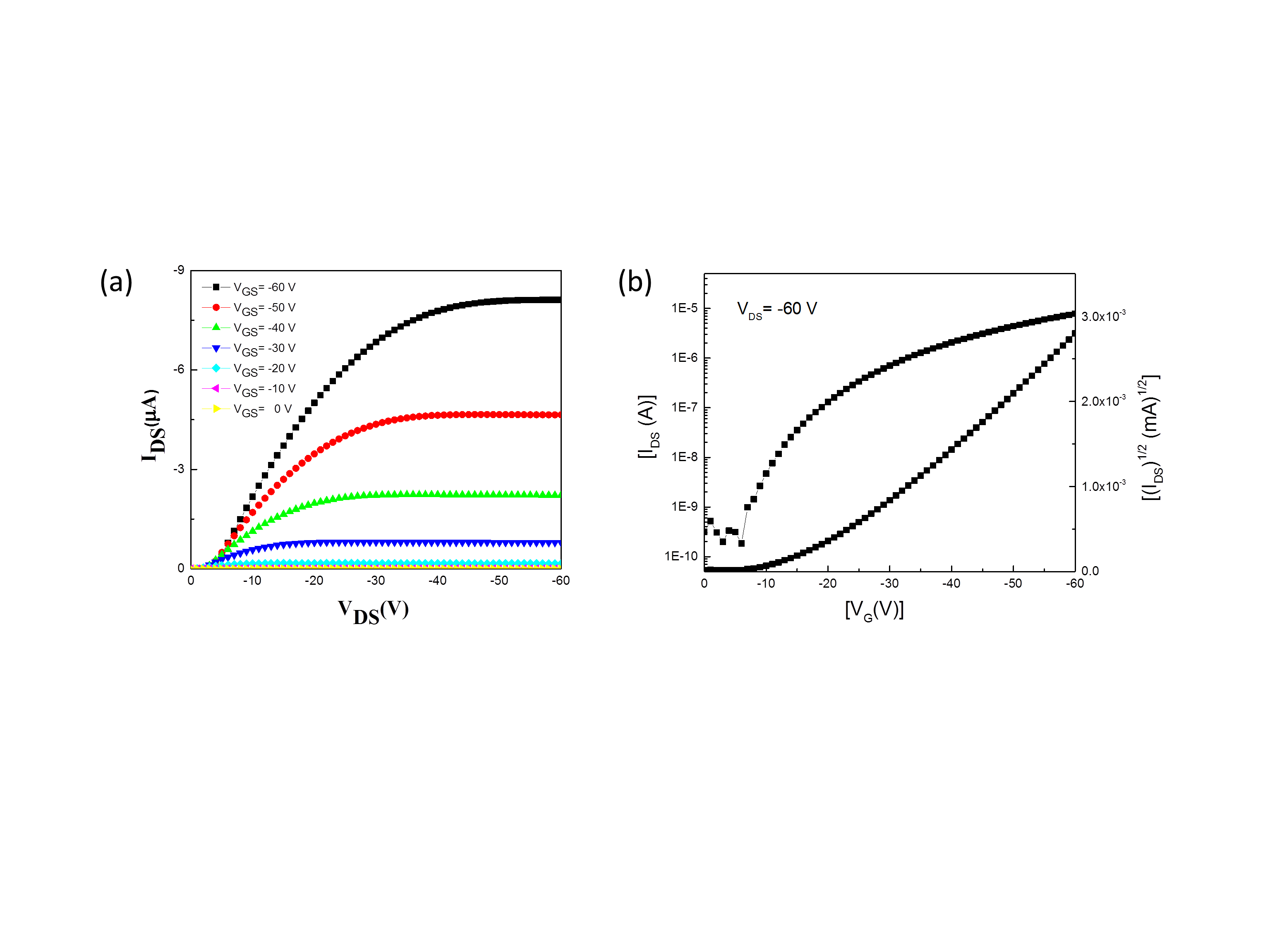


**Figure S1**. (a) Output and (b) transfer characteristics of pentacene OTFT featuring 450-nm-thick PPC as the dielectric and Au as source-drain electrode.


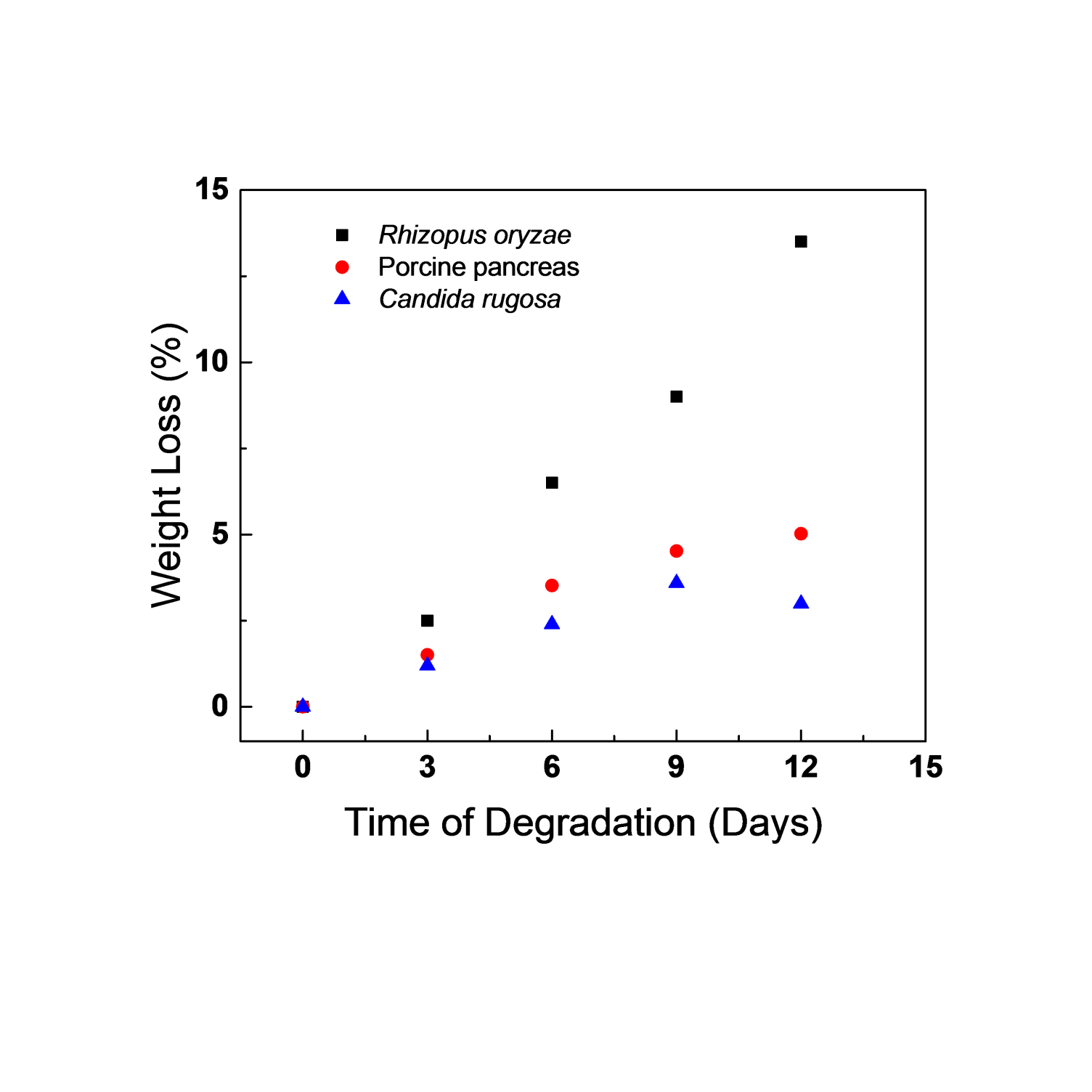


**Figure S2**. Weight losses of PPC films after immersion for 12 days in solutions of the lipases from *Rhizopus oryzae*, porcine pancreas, and *Candida rugosa*.
